# *Vibrio cholerae* at the Intersection of Immunity and the Microbiome

**DOI:** 10.1128/mSphere.00597-19

**Published:** 2019-11-27

**Authors:** Ana A. Weil, Rachel L. Becker, Jason B. Harris

**Affiliations:** a Division of Infectious Diseases, Massachusetts General Hospital, Boston, Massachusetts, USA; b Department of Medicine, Harvard Medical School, Boston, Massachusetts, USA; c Department of Pediatrics, Harvard Medical School, Boston, Massachusetts, USA; d Division of Pediatric Global Health, Massachusetts General Hospital, Boston, Massachusetts, USA; University of Missouri-Kansas City School of Medicine

**Keywords:** *Vibrio cholerae*, cholera, immune response, innate immunity, microbiome, vaccine

## Abstract

Vibrio cholerae is a noninvasive pathogen that colonizes the small intestine and produces cholera toxin, causing severe secretory diarrhea. Cholera results in long lasting immunity, and recent studies have improved our understanding of the antigenic repertoire of V. cholerae. Interactions between the host, V. cholerae, and the intestinal microbiome are now recognized as factors which impact susceptibility to cholera and the ability to mount a successful immune response to vaccination. Here, we review recent data and corresponding models to describe immune responses to V. cholerae infection and explain how the host microbiome may impact the pathogenesis of V. cholerae. In the ongoing battle against cholera, the intestinal microbiome represents a frontier for new approaches to intervention and prevention.

## Abstract (precis)

Vibrio cholerae is a noninvasive pathogen that colonizes the small intestine and produces cholera toxin, causing severe secretory diarrhea. Cholera results in long lasting immunity, and recent studies have improved our understanding of the antigenic repertoire of V. cholerae. Interactions between the host, V. cholerae, and the intestinal microbiome are now recognized as factors which impact susceptibility to cholera and the ability to mount a successful immune response to vaccination.

## INTRODUCTION

Vibrio cholerae is a highly motile, salt-tolerant bacterium. It was identified as the cause of cholera by Pacini in 1854, which was later confirmed in 1884 by Koch (1, 2). Further understanding of the pathogenesis of cholera stalled until the 1950s, when Nath De demonstrated the effects of cholera toxin (CT) by injecting rabbit ileal loops with cell extracts from cultured V. cholerae (3). While our understanding of the regulation and mechanisms of action of CT have advanced considerably, there are still significant gaps in our understanding of the pathogenesis of cholera. In this review, we highlight two such areas. First, how is protective immunity against V. cholerae generated? Second, how does the intestinal microbiome impact host-pathogen interactions in V. cholerae pathogenesis and immunity?

## ETIOLOGY

V. cholerae is a facultative pathogen. It persists in aquatic reservoirs and forms biofilms in association with plankton (4). Although environmental V. cholerae is diverse, cholera is caused by a restricted subset of pandemic V. cholerae strains which cyclically emerge and replace their precursors. The current, seventh pandemic V. cholerae biotype El Tor (7PET) lineage was first recognized as a cause of widespread cholera in 1961 and, within two decades, replaced the previous sixth pandemic classical biotype globally (5). The emergence of new dominant lineages is also apparent within the seventh pandemic, and genotyping of 7PET isolates reveals three distinct but overlapping waves of transmission, each associated with horizontal gene acquisitions (6). The SXT/R391 antibiotic resistance element was acquired during the second wave, and a new CT-encoding bacteriophage, similar to that associated with the previous sixth pandemic classical biotype, replaced the toxin encoding region during the third wave (6).

More than 200 serogroups of environmental V. cholerae are defined by their O-antigen structure (7), but only serogroup O1 is associated with pandemic cholera (8). Other serogroups have caused sporadic cases or limited outbreaks. A unique exception thus far is the V. cholerae O139 serogroup, which caused epidemic cholera from 1992 to 2002 (9). V. cholerae O139 resulted from a single horizontal gene exchange of the *rfb* (O-antigen encoding) locus in the circulating 7PET O1 strain (10). After this serendipitous recombination event, it is possible that an increase in prevalence of V. cholerae O139 was then facilitated by the niche created by widespread existing immunity to the O1 serogroup and a corresponding lack of immunity to the emergent O139 serogroup. This is conceivable given that V. cholerae O1 and O139 infections confer homologous immunity (against reinfection with the same serogroup) but not heterologous immunity (against infection with the other serogroup) (11). However, because other non-O1 strains do not typically cause cholera epidemics, it is likely that unknown constraints prevent their more frequent emergence.

V. cholerae serogroup O1 is divided into two serotypes, Inaba and Ogawa. The difference between serotypes is the absence of a single methyl group in the terminal perosamine of the O-polysaccharide in Inaba, an alteration acquired through a lack of function mutation in the *wbeT* methyltransferase (11). In areas of endemicity, either serotype may predominate for years (12). The prolonged serotype cycles can be explained by a high, but incomplete level of cross-protection between serotypes (13). This model also explains why there is a transient increase in the average age of patients with cholera that coincides with shifts in the dominant serotype (12). However, one longitudinal study in a cholera endemic area of Bangladesh suggests that cross-serotype immunity is asymmetric (14). While V. cholerae O1 Inaba infection conferred protection against both serotypes, there was no evidence of cross-protection against Inaba following V. cholerae O1 Ogawa infection (14). This differs from human challenge studies that demonstrate protection following infection with either serotype for at least 3 years (15). Considering these results, the mechanisms which generate and maintain serotype-specific immunity and serotype cycling are not fully understood (16).

## PATHOGENESIS

Cholera is a severe secretory diarrhea which can result in death within hours of the onset of symptoms (17). Fluid losses may exceed 1% of total body weight per hour (18). Infection usually requires ingestion of a large inoculum, and in North American adult volunteers, between 10^8^ and 10^11^ viable organisms are needed to produce disease consistently. This is because most V. cholerae are killed in the acidic gastric environment (18) and the required inoculum is decreased in individuals with reduced gastric acidity (19).

Once V. cholerae reaches the intestine it is propelled by a single sheathed flagellum. It then penetrates the mucus barrier to adhere to the small intestinal mucosal surface (20). Motility is required for successful colonization. In animal models of cholera, V. cholerae preferentially colonizes the mid-small intestine to the distal small intestine, where it forms clonal microcolonies in villous crypts (21). The presence of mucus, bile, and other external signals activate the ToxR regulon, a signaling hub which controls virulence through the expression of CT and the toxin-coregulated pilus (TCP) (22). All cholera-causing strains of V. cholerae harbor the ToxR regulon and the machinery to secrete both TCP and CT. TCP is a long, flexible type IV pilus that is required for colonization (23). It is made up of a repeating configuration of TcpA, its main structural subunit (24). TCP is also the receptor for the lysogenic bacteriophage CTXϕ, which encodes CT, an AB_5_-subunit toxin (24). CT is composed of one enzymatically catalytic A subunit (CtxA) and a pentamer of B subunits (CtxB). The B subunit pentamer binds the monosialoganglioside GM1 via cell surface receptors on the apical surface of the epithelium (23, 25). The toxin is endocytosed, and CtxA escapes the endosome to ribosylate the G-protein-regulated adenylyl cyclase on the cell basolateral membrane (26). This results in chloride (Cl^–^) loss and massive fluid secretion into the small intestine, overwhelming the resorptive capacity of the large intestine and resulting in severe watery diarrhea (7).

The diarrhea produced by V. cholerae is a vehicle for transmission. Once V. cholerae populations in the small intestine grow to a high density, the organisms detach from the intestinal surface to escape from the host (27). In severe cholera, up to 10^9^/viable organisms are excreted per ml of stool and vomitus (28), and without effective antibiotic treatment, the secretion of organisms continues for several days (29). Organisms remain highly infectious for up to 24 h after excretion from the host, and human-shed organisms have higher infectivity compared to aquatic V. cholerae. This increased infectivity may contribute to spread during cholera epidemics (30).

## INNATE IMMUNITY

Unlike invasive intestinal bacterial pathogens like *Shigella* and *Salmonella*, V. cholerae infection does not cause clinically overt inflammation. Nonetheless, cholera disrupts the mucosal barrier at a microscopic level, resulting in widening of intracellular spaces, disruption of the apical junction, and an influx of neutrophils, macrophages, and other lymphocytes into the lamina propria (27, 31, 32). Cholera also triggers the production of innate effector molecules at the mucosal surface, such as lactoferrin, defensins, and oxidase (30, 31, 33, 34). In severe cholera, the disruption of intestinal homeostasis lasts for up to 6 months (33), much longer than the diarrheal symptoms of cholera, which usually resolve within an average of 2 to 4 days depending on antibiotic treatment (35).

V. cholerae infection activates several hubs of innate immune signaling (30, 33). This has been observed in biopsy specimens from patients with acute cholera and can be modeled *in vitro* (30, 33, 36, 37). Some of the major innate signaling pathways which are upregulated in response to V. cholerae are not typical of the innate immune response to bacterial infection. For example, both the NLRP3 inflammasome and type I interferon signaling pathways are highly activated in response to cholera, even though such responses are canonically associated with responses to viral infection (33).

Activation of the host-innate immune system in cholera may serve more than one purpose, but whose purpose; the host or the pathogen? Innate immunity serves as a first line of defense, but in severe cholera this initial defense is clearly ineffective. In the short term, the human-innate immune response may better serve the pathogen, since the production of innate effectors to which V. cholerae is resistant may provide an edge in its competition with commensal organisms. For example, innate signaling pathways activated during V. cholerae infection induce the expression of proteins that generate reactive oxygen species. Dual oxidase 2 (DUOX2) and inducible nitric oxide synthase (iNOS) are both among the most upregulated proteins in duodenal tissue during cholera (30, 33). However, the antibacterial effects of the resulting reactive oxygen species may end up benefitting V. cholerae in its competition with gut commensals, since V. cholerae employs inducible resistance to oxidative stress during colonization (38, 39).

In addition to its role in immediate defense, the innate immune system also directs the development of subsequent adaptive immunity. The innate immune system senses pathogen and damage associated molecular patterns, then produces signals which direct T and B lymphocyte responses. This immune modulation is achieved through the production of cytokines, costimulatory molecules, and other signals. In cholera, the production of cytokines, including interleukin-1β (IL-1β), IL-6, and IL-17, are increased in response to acute infection (33, 40). This role of innate immunity in shaping the adaptive response may also explain why variations in the type I interferon and inflammasome signaling pathway which are associated with susceptibility to cholera have been under strong selection pressure in Bangladesh, an area where cholera has been historically endemic (36). This is akin to the finding that individuals with blood type O are more susceptible to severe cholera, an association that may account for the low prevalence of the O blood type in the Bengal delta (41, 42).

## ADAPTIVE IMMUNITY

Infection with V. cholerae results in long-lasting immunity (14, 15, 43). Human challenge studies suggest that complete protection lasts at least 3 years, which is the longest interval tested (15). Surveillance in Matlab, Bangladesh, from 1968 to 1977 showed that an episode of cholera resulted in 90% protection against subsequent disease over the entire follow-up period. In this cohort, there were only three repeat hospitalizations from cholera out of a predicted 30, all of which occurred in young children (43). Similarly, mathematical models based on decades of longitudinal surveillance in areas of endemicity suggest that the level of immunity remains stable for 5 years or more after infection (13).

## ANTIGEN REPERTOIRE

**FIG 1:**
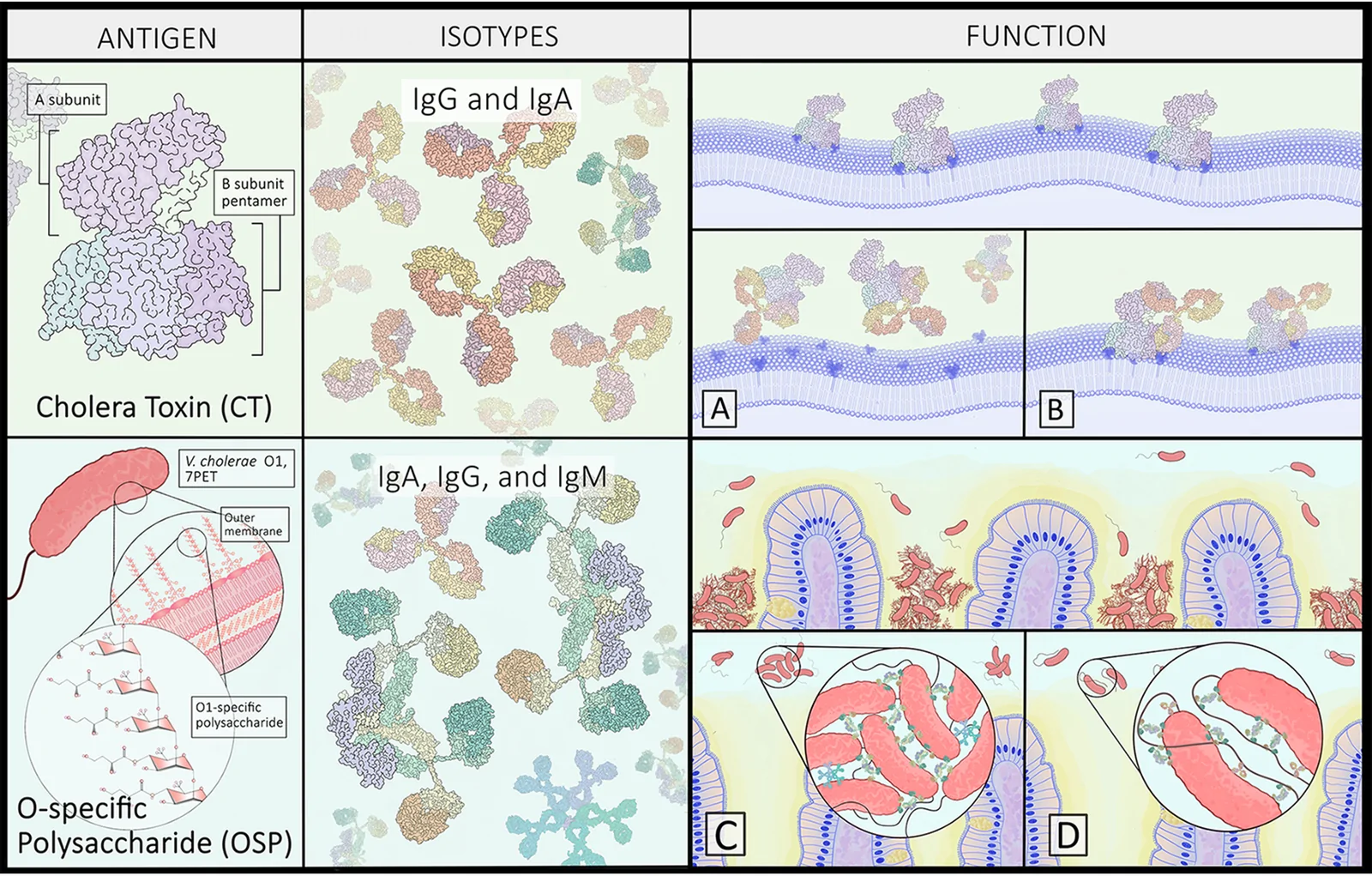
Cholera toxin (CT) and the O-specific polysaccharide (OSP) are the two dominant V. cholerae antigens. Remarkably, following infection in an area of endemicity, more than 75% of the antibodies derived from clonally expanded plasmablasts targeted either CT or the OSP. CT antibodies target both the A and B subunits and may block toxin binding (A) or activity (B), yet the persistence of circulating antitoxin antibodies does not appear to confer long lasting immunity to cholera. OSP-specific antibodies target the bacterial outer membrane and confer protection. Several mechanisms have been proposed, including agglutination (C) and motility inhibition (D) or other effector functions which may entrap V. cholerae before it reaches the mucosal surface, such as activation of neutrophil extracellular traps.

V. cholerae produces many potential antigens, but only a few appear to be dominant targets of human immunity (Fig. 1). This was demonstrated by a 2016 study which evaluated the antigenic repertoire of recombinant monoclonal antibodies (MAbs) generated from individually sorted plasmablasts from Bangladeshi patients recovering from cholera (44). Over 75% of the antibodies produced from clonally expanded plasmablasts bound to CT or the O-specific polysaccharide antigen (OSP) (44). Additional screening of the MAbs using a V. cholerae proteome array demonstrated that the sialidase NanH (which facilitates toxin binding by converting higher-order cell surface gangliosides to GM1) was a third dominant antigen, though to a much lesser extent than CT and OSP (44). Other known antigens include TcpA, hemolysin A, and flagellar proteins (44, 45). In the case of TcpA, repeated exposure appears to be required to prime class-switched responses (46).

## ANTITOXIN RESPONSES

CT is the primary virulence factor of V. cholerae and ingestion of as little as 5 μg produces the symptoms of cholera (47). Despite this, antitoxin responses do not appear to mediate long-term protective immunity against cholera. One limitation of previous studies of antitoxin immunity has been their persistent focus on the GM1-binding CtxB subunit. Previous studies focused on CtxB, because it was found to be the immunodominant component of CT in animal models of cholera, and because this nonactive component of the toxin was an obvious vaccine-antigen candidate (48, 49). A study of responses in CT-immunized mice demonstrated that CtxB-specific responses were dominant and protective while CtxA-binding antibodies had no appreciable toxin-neutralizing capacity (50). However, in humans, CtxB-binding antibodies do not appear to confer durable protection. This is supported by the fact that circulating levels of CtxB-IgG antibodies and CtxB-specific memory B cells are not associated with protective immunity in household contacts of patients with cholera, nor in human challenge studies (51–53). Although elevated circulating levels of CtxB-specific IgA are associated with protection, these wane within months after natural infection (53, 54).

These findings are in line with observations from cholera vaccine trials. In one major field trial, while the addition of CtxB to inactivated oral whole-cell vaccines briefly boosted the effectiveness of vaccination (compared to the inactivated vaccine without CtxB), it did not improve protection beyond the first 8 months. This suggests that CtxB provides only a short-term boost of immunity (55), and more concerningly, that overall protection with the CtxB containing vaccine appeared to drop to lower levels than the non-CtxB containing vaccine within 2 years after vaccination (56). This reduction in long-term immunity may be due to the immunomodulatory effects of CtxB (40).

Similarly, trials of oral vaccination with a glutaraldehyde-treated CT toxoid vaccine alone produced only limited short-term immunity (57). But why do anti-CT responses fail to provide lasting protection against a singular toxin mediated disease? High levels of CtxB-specific sIgA at the mucosal surface are present for months after infection and may provide protection in the short term. However, when exposure occurs more distantly from initial infection, there may be insufficient time to mobilize an effective anamnestic antitoxin response at the mucosal surface once bacterial toxin production is already established.

Still, it may be worth reevaluating the role of CtxA as a protective antigen. This is because recent studies show that unlike mice exposed in a laboratory, humans living in areas where cholera is endemic have prominent CtxA-specific antibody responses (44). More importantly, CtxA antibodies are capable of neutralizing toxin activity at very low concentrations. Because CtxA-antibodies are highly cross-reactive with the enterotoxigenic Escherichia coli (ETEC) heat-labile toxin (LT) (44) and because exposure to the ETEC LT toxin is a frequent occurrence for people living where cholera is endemic, it is likely that the CtxA response observed following human cholera is the result of the expansion of cross-reactive memory B cells derived from prior ETEC infection (44). This may explain why humans respond differently to CtxA than laboratory mice and is an excellent example of the limitations of animal models in studies of human adaptive immunity, where exposure to other infections and commensal organisms may dramatically shape the immune response.

## ANTIBACTERIAL RESPONSES

Unlike antitoxin responses, functional antibody responses directed at the bacterial outer membrane O-polysaccharide are more overtly important in protection against cholera. The serum vibriocidal antibody titer has been used to measure the functional humoral immune response to V. cholerae for decades; it remains the best seroepidemiologic marker of recent exposure (58), and the best-established immunologic correlate of protection against cholera (53, 59, 60). The vibriocidal titer measures the lowest concentration of serum or plasma required for antibody-dependent complement mediated bacterial killing, and vibriocidal antibodies almost exclusively target OSP (44, 61).

Despite the utility of the vibriocidal antibody titer as a correlate of protection, it is unlikely that protection is mediated by a circulating, complement-fixing antibody. First, although increasing vibriocidal titers are associated with protection against cholera, there is no threshold titer at which 100% protection is achieved (62). Second, while complement mediated killing is essential in protecting against certain bloodborne pathogens, it is unclear whether there is enough complement at the mucosal surface to block colonization with V. cholerae. Third, at the intestinal surface, sIgA responses predominate, and sIgA does not induce complement activation. A caveat to this conclusion is that, in areas where cholera is endemic, class switched OSP responses also induce IgM and IgG memory B cell responses (44, 63). Thus, complement mediated killing is not completely excluded as a possible defense against V. cholerae.

Given that motility is required for colonization, inhibition of bacterial motility has been proposed as a mechanism of protection against cholera, and OSP-targeted IgA antibodies can directly inhibit motility by interfering with flagellar function (64–66). Other proposed antibody-mediated mechanisms include bacterial trapping and clearance prior to penetration of the mucous barrier and colonization of the small intestine (16, 67, 68).

Another important question is how are OSP-specific antibody responses maintained? One possibility is that immunity is maintained by long-lived plasma cells at the mucosal surface. However, basal levels of OSP-specific intestinal sIgA secretion drop quickly after recovery from cholera (69). Another possibility is that mucosal antibody-mediated immunity is maintained in the memory B cell compartment (16, 70). Memory B cells express antibodies at the cell surface but do not secrete them. Yet, memory B cells are capable of rapid differentiation into plasma cells and generation of anamnestic immune responses upon reexposure to antigen.

There is already evidence that the presence of circulating OSP-specific memory B cells is associated with protection against cholera, even in individuals with undetectable levels of circulating vibriocidal antibodies (52, 71, 72). This association has been observed in household contacts of patients with cholera (71, 72) and in recipients of an attenuated V. cholerae vaccine who were challenged with wild-type V. cholerae (52). In vaccinees, the magnitude of the initial vibriocidal antibody response was strongly predictive of their subsequent OSP memory B cell response (52). Interestingly, while mucosal O-antigen responses are thought to occur primarily through T-cell-independent IgA class switching pathways, V. cholerae O antigen responses in patients recovering from cholera are characterized by high levels of somatic hypermutation, affinity maturation, and cross-reactive recall responses of memory B cells from prior antigen exposure (44). These features of the OSP response are evidence that long-lasting memory B cell responses may play a role in maintaining immunity against cholera.

## THE MICROBIOME AND CHOLERA

The microorganisms composing the complex and self-regulated community of the gut microbiota are increasingly recognized as an important factor in enteric infections. Advances in metagenomic profiling of microbial communities are beginning to reveal the physiologic mechanisms of microbiome-related effects on enteric infection that have been appreciated for decades. These include resistance to colonization and the anti-bacterial activity of commensal gut microbes. For example, in cholera, it has long been recognized that infection in animal models requires disruption of the commensal microbiota with antibiotics, because the undisturbed gut microbiota is otherwise protective against V. cholerae infection (73, 74).

Our understanding of how gut microbes impact host-pathogen interactions in V. cholerae infection is nascent. Because presence of specific members of the gut microbiota correlate with susceptibility to cholera, further studies investigating causality and mechanisms behind this association may identify new approaches to prophylaxis and treatment. Cutting-edge studies of how the microbiota modulate immune responses to oral cholera vaccination may also impact prevention.

## EFFECT OF CHOLERA ON THE MICROBIOME

Cholera essentially eradicates the normal gut microbiota (75). First, the massive efflux of water into the intestinal lumen washes away the protective mucus where much of the gut microbiota resides. Second, depletion of the microbiota is likely exacerbated by treatments for cholera, including ingestion of large amounts of oral rehydration solution and antibiotics that kill some gut bacterial species (75, 76). Based on DNA sequencing of all bacteria in the stool during the initial phase of infection, V. cholerae itself makes up the majority of bacteria found in rice-water stool (75, 77). Alterations in the microbiota extend beyond the duration of symptoms, and recovery after infection follows a distinct pattern (75). Colonizing gut microbes are scant immediately following infection. Then, in the first few days of recovery, aerobes and facultative anaerobes ingested from the environment and the oral cavity dominate the microbiota. These recolonizing organisms likely flourish due to the abundance of nutrients and oxygen that accumulates during the early recovery period while the flora is depleted. As aerobes proliferate, oxygen tension is lowered, and obligate anaerobes again colonize the gut. Several weeks after cholera, community profiling demonstrates a return to a near-baseline gut microbiota composition, when competition for nutrients and resources resumes (75). A similar pattern of recovery was also demonstrated in a study of gut microbiota post-ETEC infection in Bangladesh, although in an ETEC human challenge study, anaerobic species persisted during acute infection in some subjects (75, 78). The dissimilarity in patterns of microbial recovery in these two groups could be due to differences in baseline microbiota of American volunteers compared to Bangladeshis. Alternatively, these differences may exist due to factors that we do not yet understand regarding underlying patterns of microbial recovery after acute diarrhea. The disruption to gut homeostasis after cholera and other forms of severe diarrhea is associated with enteric dysfunction and malnutrition in children living in areas of inadequate safe water sources (79, 80). While the gut microbiota likely mediates some of this pathology, this complex relationship and the physiology of enteric dysfunction remains poorly understood (81, 82).

## THE MICROBIOME AND *V. CHOLERAE* PATHOGENESIS

Household contacts of patients with cholera are at high risk for infection. In a prospective evaluation of household contacts of patients with cholera, there was a strong association between specific microbial groups at the time of exposure to V. cholerae and infection during the following week. In fact, the composition of the gut microbiome predicted infection as well as the known clinical, immunologic, and epidemiologic risk factors for cholera (83). Based on a machine learning model, bacterial taxa were ranked according to their association with either increased or decreased susceptibility to infection, and the top 100 bacterial taxa from this ranking successfully discriminated between clinical outcomes. However, the baseline alpha and beta diversity alone were not predictive of susceptibility to cholera. This contrasts with other enteric pathogens which are more likely to cause infection in humans with a lower-diversity microbiome (84, 85). Investigation of the specific bacterial groups correlated with susceptibility may provide further insight on relationships between V. cholerae infection, the gut microbiome, and clinical outcomes.

### Mucins, bile acids, and T6SS.

**FIG 2:**
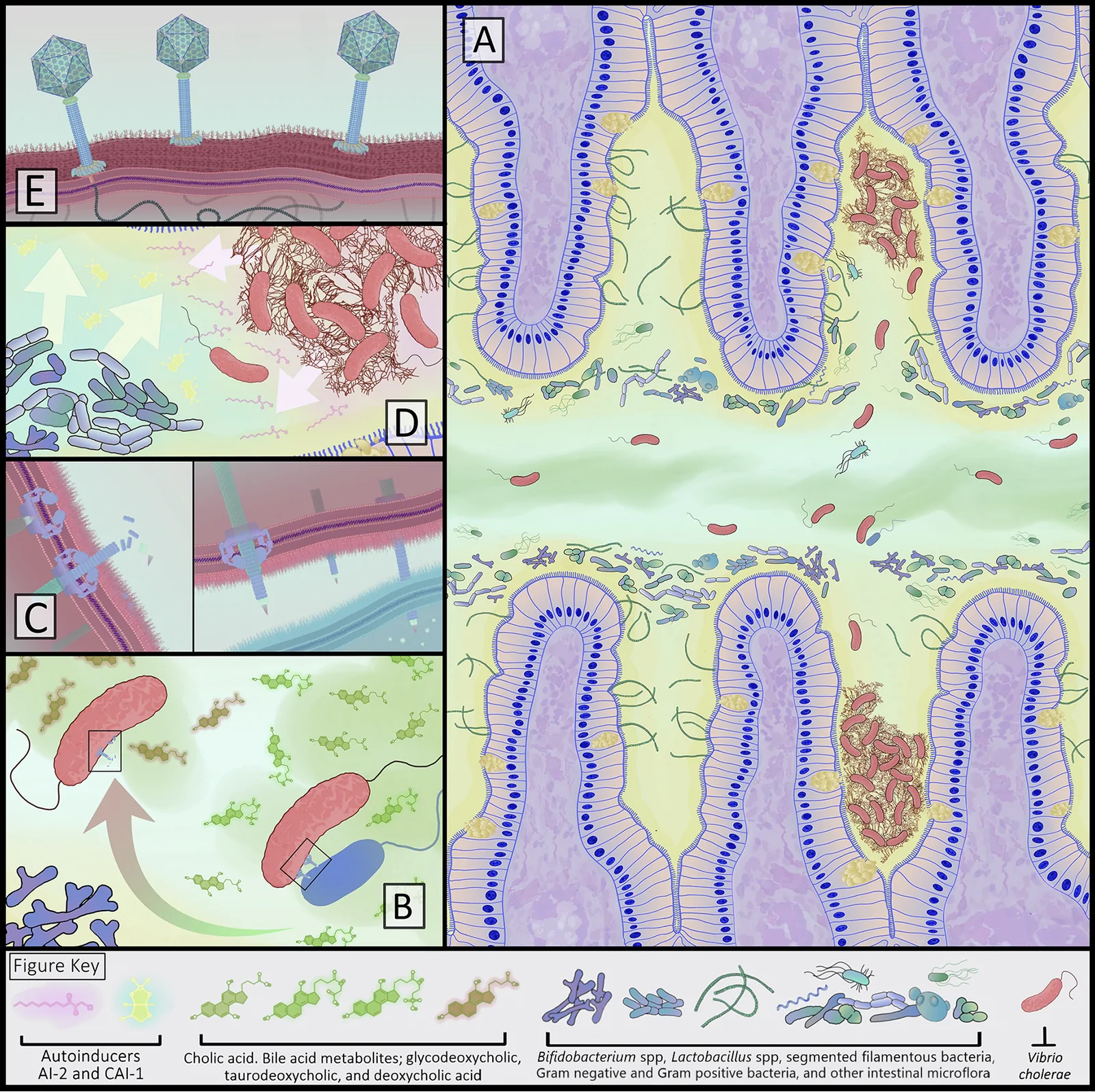
Interactions between the gut microbiota, their metabolites, V. cholerae, and the small intestinal environment. (A) After V. cholerae survives the acidic gastric environment, the pathogen enters the small intestinal gut lumen, where both bile (green) and mucus (yellow) signal to V. cholerae to express the virulence factors that cause symptomatic infection. Mucus coats the villi and acts as a diffusion barrier, and V. cholerae uses flagellar motion to traverse the inner and outer mucus layers. During this journey V. cholerae encounters the resident gut microbes and their metabolites. After reaching the intestinal epithelial crypts, V. cholerae forms biofilms (shown as fibrous mats of organisms) to adhere to the epithelial surface. (B and C) When encountering the mucus layer, the V. cholerae type VI secretion system (T6SS) is activated. This system operates as a contractile organelle that extends from V. cholerae to make contact with neighboring organisms to translocate toxic effectors. T6SS activity can be suppressed by metabolites of cholic acid formed by gut microbiota that process bile (green and red curved arrow). (D) Autoinducer AI-2 (yellow) is produced by some commensal gut microbes and can induce quorum-sensing responses in V. cholerae. The presence of autoinducers indicate to V. cholerae a high density of organisms, resulting in reduced expression of virulence genes that enable colonization and cholera toxin production, and activation of genes that promote exit from the host, such as increased flagellar motion. (E) Bacteriophage specific to V. cholerae (vibriophage) can infect and lyse large numbers of organisms rapidly, drastically reducing V. cholerae populations.

Sensing of the intestinal environment allows V. cholerae to tailor the expression of virulence and compete with the host microbiota for resources, including access to the epithelial surface. In colonizing the small intestine, V. cholerae encounters a layer of highly glycosylated proteins (mucins) lining the intestinal epithelium (Fig. 2). This mucus layer is also heavily colonized with commensal bacteria, and V. cholerae detects the presence of these microbes and their antibacterial metabolites through various mechanisms. Sensing of mucin activates the V. cholerae type VI secretion system (T6SS), which operates as a molecular syringe, delivering toxic proteins to other bacteria (86, 87). In a study screening for genes critical to *in vivo* V. cholerae fitness, the T6SS apparatus was found to be vital for successful colonization, highlighting one method by which V. cholerae overcomes colonization resistance (88). In a study of mice infected with V. cholerae T6SS mutants compared to wild-type strains, V. cholerae colonization was increased several fold in wild-type infections due to the T6SS-mediated attack of host commensal organisms (89).

Another aspect of the small intestinal environment that impacts this interaction is cholic acid from bile, which can be processed by some gut microbes. V. cholerae recognizes bile acids as a cue for the small intestinal environment, and upon exposure to bile acid, gene expression shifts to optimize locating the mucosal epithelium through increased motility while repressing virulence factors CT and TCP (90). Some small intestinal microbes also dehydroxylate primary bile acids, and when V. cholerae senses the metabolic product deoxycholic acid, T6SS activity is suppressed (86). By metabolizing bile acids and masking the “sensing” of the small intestinal environment, these commensals may advance their own survival. Through the enterohepatic circulation in the liver, both primary and secondary bile acids can be conjugated, resulting in metabolites that enhance T6SS function (86). Sensing of bile and bile acid metabolites by V. cholerae impacts the infected host because CT and TCP expression vary depending on the specific form of bile acid present in the small intestine (91, 92). For further resolution on how V. cholerae fine-tunes virulence expression based on the intestinal environment, we need a better understanding of the gut microbes that participate in bile acid metabolism and how V. cholerae virulence is impacted by these metabolites.

### Autoinducers.

Small molecules produced by the gut microbiota and detected by V. cholerae are an example of interspecies communication that impacts pathogenesis. Two quorum-sensing molecules, autoinducer-2 (AI-2) and cholera autoinducer 1 (CAI-1) are sensed by V. cholerae using histidine kinase receptors LuxQ and CqsS, respectively (93). Recognition of autoinducers allows for population-level coordinated activity by V. cholerae. When V. cholerae is present at high density, a quorum is “sensed” and autoinducers are produced (94). Autoinducer binding results in modulation of downstream virulence factors, including a reduction in TcpA expression and production, which signals that V. cholerae should disassociate from the epithelial surface (93). Thus far, the autoinducer CAI-1 is known to be produced naturally only by *Vibrio* species. In an infant mouse model of V. cholerae infection, an engineered strain of E. coli made to express CAI-1 resulted in an 80% reduction in CT binding at the intestinal surface, thereby preventing V. cholerae colonization (95).

In humans recovering from cholera, AI-2 production by commensal intestinal bacteria was found to block V. cholerae virulence expression (77). In a 2014 study, a community of 14 gut microbes associated with recovery from human cholera was reconstituted in germ-free mice. Upon challenge with V. cholerae, one species, Blautia obeum (formerly Ruminococcus obeum), was associated with a reduction in V. cholerae colonization through a LuxS-based AI-2-dependent signaling pathway. This demonstrates one mechanism of host gut microbe colonization resistance through disruption of V. cholerae quorum sensing via interspecies signaling (77). Because AI-2 is made by numerous members of the gut microbiota, this may represent one of many examples of interspecies communication that impact virulence (96, 97). Studies in humans are needed to determine whether autoinducers from naturally occurring host gut microbes or engineered species could impact clinical outcomes.

### Other gut microbe metabolites.

Commensal bacteria have long been postulated to influence behavior of V. cholerae by secreting antimicrobial compounds, and these interactions have been studied by applying culture supernatant from commensals to *in vitro* V. cholerae culture or in animal models of infection. Prior to the advances of genomic analyses, plating of fecal samples from patients with cholera demonstrated restriction of V. cholerae growth in the presence of *Lactobacilli* and *Peptostreptococcus* species due to unidentified “inhibitory diffusible compounds” (98). In a 2018 study, *Lactobacillus* species from the stool of healthy children were screened to detect effects on formation and dispersal of V. cholerae biofilms (99). Biofilms are an important virulence factor for V. cholerae survival, facilitating adherence to the intestinal epithelium, protecting the pathogen from antibiotics and acid inactivation, and even protecting against predation by other gut microbe species (100). Metabolites in the culture supernatant of seven *Lactobacillus* isolates inhibited V. cholerae biofilm formation in a pH-dependent manner, although the structure and function of the antimicrobial compounds in these studies remain unknown (99).

Several other known microbial metabolites can alter the chemical environment, impacting V. cholerae pathogenesis. In infant mice, ingestion of microbes that secrete lactic acid reduce V. cholerae growth and colonization (101). Like many bacteria, V. cholerae is also sensitive to reactive oxygen species. Infant mice that ingest commensal E. coli strains defective in ROS degradation prior to V. cholerae challenge had a higher susceptibility to infection compared to those given E. coli with the ability to degrade reactive oxygen species (102). While these studies exemplify how a single metabolite or environmental shift can potentially alter the course of infection, the relevance of these findings in human cholera is not known. Based on the recognized mechanisms of microbial metabolites that interact with V. cholerae and the complexity of the bacterial community of the gut, the full scope of metabolites that impact V. cholerae pathogenesis are likely heterogeneous in structure and mechanism.

### Vibriophage.

In addition to bacteria, the intestine harbors fungi, parasites, and viruses, including bacteriophage. While hundreds of bacteriophages are known to infect V. cholerae, few have been characterized (103). Vibriophage are found in humans recovering from cholera, and phage predation of V. cholerae has been observed *in vivo* during human infection, where it is associated with the rapid acquisition of intrahost V. cholerae mutations in phage receptors (104). This suggests that lytic vibriophage has the potential to impact the course of disease in humans. In the preantibiotic era, two studies evaluated the efficacy of vibriophage administration for treatment of cholera in humans (105, 106). In India, a reduction in cholera cases was also observed after adding vibriophages into community drinking water on a weekly basis (106). However, because V. cholerae resistance to lytic phage evolves rapidly (102, 104, 107), it is likely that combinations of vibriophages would be required for effective treatment of clinical V. cholerae strains.

## THE GUT MICROBIOME AND ORAL CHOLERA VACCINE RESPONSES

Both live-attenuated and killed oral cholera vaccines (OCV) are increasingly used to prevent cholera (108, 109), and killed OCVs are a critical component of the World Health Organization’s strategy to reduce the global threat of cholera by 2030 (110). Because OCVs are absorbed at the mucosal surface where the intestinal microbiota continually interfaces with immune cells conducting antigen surveillance, the gut microbiome could impact immune responses to vaccination. This has led to the hypothesis that the gut microbiota is a frequently unmeasured host factor that partially determines immune responses to OCV. Evidence supporting this theory dates to the 1990s when bacterial overgrowth was found to correlate with reduced response to an attenuated OCV (111).

While the gut microbiota’s impact on OCV responses has not yet been systematically studied, emerging data from other oral vaccines suggest relevant interactions (112). For example, the immunogenicity of oral live-attenuated typhoid vaccine in adults was correlated with more diverse gut microbial communities, and the abundance of several specific microbes differentiated multiphasic from late cell-mediated immune responses after vaccination (113). A stool analysis of pairs of infants from Ghana and the Netherlands compared discordant immune responses to oral rotavirus vaccination and showed that the gut microbiome of responders was similar regardless of country of residence, and that nonresponders from either country had less Streptococcus bovis and increased organisms from the phylum *Bacteroidetes* (114). Although little studied, the nonbacterial microbial factors impacting baseline immunoactivation or inflammation may also impact immune responses to OCVs. For example, children receiving antihelminth treatment prior to vaccination had higher vibriocidal responses to OCVs compared to untreated children (115). However, antihelminth treatment did not improve immune responses of Bangladeshi children to Ty21a, an oral typhoid vaccine (116). Use of perivaccination antibiotics is an additional microbiota-altering intervention that could impact immune responses to OCVs, and this has yet to be studied systemically. Further investigation is needed to understand the dynamics and advantages of adjunctive treatments that affect the gut microbiota at the time of vaccination.

To date, studies of oral vaccination in humans and the gut microbiota have not evaluated mechanistic hypotheses or explored causative relationships. Yet, based on current knowledge of how gut microbes can impact human immune responses in the gut, it is clear that specific metabolites of the gut microbiota have the potential to impact immune responses to OCVs. For example, short-chain fatty acids (SCFAs) are by-products of nondigestible carbohydrate metabolism by some members of the anaerobic microbiota and are known to impact the development of mucosal immune responses in other disease processes (117, 118). The effect of SCFAs on OCV responses has thus far only been tested in cell culture. When the SCFA butyrate was added to cultured gut epithelial cells and exposed to an attenuated V. cholerae vaccine, CCL20 production was increased (119), providing a pathway by which butyrate may accentuate mucosal immune responses by recruiting dendritic cells and lymphocytes. Specific SCFAs are also known to depress the effect of LPS-induced cytokines on the development of T cell subgroups, although these possibilities have not been investigated in V. cholerae infection or vaccination (120, 121). SCFAs produced in the normal gut microbiota in mouse models increased CT specific antibody responses, and these high-level antibody responses were restored to antibiotic-treated mice fed acetate and butyrate (122). However, it is not yet clear whether SCFAs impact responses to V. cholerae antigens more closely associated with protection from cholera in humans, such as OSP. Further studies that include testing of mechanistic hypotheses are needed to advance the potential for microbes (or their metabolites) to be used as vaccine adjuvants.

### Probiotic impact on *V. cholerae* infection and OCVs.

Probiotics have safely been coadministered with OCVs in two small, randomized placebo-controlled trials without a clear effect on vaccine immunogenicity (123, 124). These studies and others evaluating the effect of probiotics on human health are hampered by variation in choice of bacterial strain, purity, dose, and timing of administration, limiting comparisons between studies (125). The challenge of interpreting cause and effect is further compounded in studies that do not account for human host factors known to impact immune responses to OCVs, such as age. In addition, whether a probiotic strain is just “passing through” or whether sustained colonization is necessary to effectively modulate OCV responses is an important unanswered question. For further development of probiotics as OCV adjuvants, identification of an appropriate strain in addition to optimization of the dose and formulation are needed. If safety data are reassuring, testing of a probiotic OCV adjuvant should include young children and infants who are most in need of improved responses to OCV.

## ON THE HORIZON

Expansive cholera outbreaks and the large burden of cholera in areas of endemicity represent ongoing public health crises. In Yemen alone, over 1.7 million cases of cholera have occurred since 2016 (126). A better understanding of immunity, including improved biomarkers of protective immunity, is required to develop and test improved OCV candidates. Key unanswered questions include how OSP-specific antibody responses are maintained, and how the gut microbiome at the time of vaccination or infection may influence these long-term protective responses. An improved knowledge of host-pathogen interactions is needed to harness the natural phenomenon of colonization resistance and move new vaccine and therapeutic possibilities forward. Central to this goal is a need for mechanistic studies to understand how other microbes and their signals impact V. cholerae virulence expression and the development of protective immunity.

Innovative approaches for prevention may exploit colonization resistance through the administration of engineered commensal organisms. As discussed, genetic manipulation of E. coli with the ability to express CAI-1 was successful in preventing cholera in an animal model (95). In a 2016 study, a recombinant E. coli strain that expressed glycosyltransferases mimicking ganglioside GM1, the binding site for cholera toxin, absorbed cholera toxin with high avidity and resulted in survival of challenged infant mice (127). As a therapeutic possibility, vibriophages are interesting because bactericidal activity is targeted, thereby limiting the potential for the emergence of antibiotic resistance. Historical data from the preantibiotic era suggests this approach could be promising, but modern trials and human data are lacking. While interest in bacteriophage therapy has increased with the use of phage against multidrug resistant bacterial infections (128), and a triple vibriophage cocktail was found to prevent colonization and limit the emergence of antibiotic resistance in an infant mouse model of cholera (107), there are no current human trials of vibriophage or engineered microbes for cholera treatment or prevention.

Perhaps the most promising new approach to prevention combines the long-term potential of live attenuated oral cholera vaccination with the immediate impact of colonization resistance. In a 2018 study, an attenuated V. cholerae strain protected against cholera in an infant rabbit model within 24 h of administration, prior to the development of an adaptive immune response (129). This attenuated strain conferred protection in colonized infant rabbits and prevented colonization of wild-type V. cholerae, presumably by occupying the intestinal niche of the wild-type strain or by dominating resources needed for colonization. Interestingly, the mechanism of this competitive exclusion remains unknown and was not due to one specific mutation generated in the attenuated strain (129).

Overall, these studies all demonstrate that manipulating the gut microbiota to alter the course of human cholera is within reach. Ultimately a successful approach may rely on attenuated V. cholerae vaccine strains or naturally occurring commensal organisms to either provide resistance to infection or boost the effectiveness of the cholera vaccines through interactions with the host immune system. Further mechanistic studies to explore the how the human microbiome impacts immune responses to both live attenuated and killed oral cholera vaccines are needed, and insights into the mechanisms of colonization resistance are also critical for driving these new translational approaches forward.
